# Rudiments of Modern Medical Electricity

**Published:** 1901-05

**Authors:** 


Rudiments of Modern Medical Electricity. Arranged in the Form of Questions and Answers. Prepared Especially for Students of Medicine. By S. H. Monell, M. D., New York, Professor of Static Electricity in the International Correspondence Schools; Founder and Chief Instructor of the New York School of Special Electro-Therapeutics. 12mo. pp. 165. New York: Edward R. Pelton, 19 E. 16th St. 1900. [Price, $1.00.]

There is undoubtedly a demand for such a book as Dr. Monell has attempted to write—one which will present the rudiments of modern medical electricity. Most physicians know far too little of the possibilities of electricity as a therapeutic agent, and next to nothing of its practical application. Dr. Monell is competent to write a book which would supply this confessed need. But why any man fitted to write an instructive volume on any subject should choose to present it in the form of a catechism is past finding out. The book must have great merit which can survive such a style.

